# Online Spatial and Temporal Calibration for Monocular Direct Visual-Inertial Odometry

**DOI:** 10.3390/s19102273

**Published:** 2019-05-16

**Authors:** Zheyu Feng, Jianwen Li, Lundong Zhang, Chen Chen

**Affiliations:** Information Engineering University, Zhengzhou 450001, China; von9604@gmail.com (Z.F.); zhangldxd@163.com (L.Z.); gzxbcc@163.com (C.C.)

**Keywords:** visual-inertial odometry, direct approach, online calibration, spatial-temporal parameters

## Abstract

Owing to the nonlinearity in visual-inertial state estimation, sufficiently accurate initial states, especially the spatial and temporal parameters between IMU (Inertial Measurement Unit) and camera, should be provided to avoid divergence. Moreover, these parameters are required to be calibrated online since they are likely to vary once the mechanical configuration slightly changes. Recently, direct approaches have gained popularity for their better performance than feature-based approaches in little-texture or low-illumination environments, taking advantage of tracking pixels directly. Based on these considerations, we perform a direct version of monocular VIO (Visual-inertial Odometry), and propose a novel approach to initialize the spatial-temporal parameters and estimate them with all other variables of interest (IMU pose, point inverse depth, etc.). We highlight that our approach is able to perform robust and accurate initialization and online calibration for the spatial and temporal parameters without utilizing any prior information, and also achieves high-precision estimates even when large temporal offset occurs. The performance of the proposed approach was verified through the public UAV (Unmanned Aerial Vehicle) dataset.

## 1. Introduction

The monocular visual-inertial system, which is usually composed of a low-cost MEMS (Micro-electro- mechanical Systems) IMU and a camera, has turned out to be a highly attractive solution for motion tracking and 3D reconstruction due to its lightweight characteristics of size, weight and power. As a result, monocular visual-inertial state estimation has become a highly active research topic in robotics and computer vision communities.

In the last few decades, there have been a great deal of scholarly work on monocular visual-inertial state estimation. Researchers make use of IMU measurements and monocular camera observations to recover carrier motion and 3D structure. The solutions can be divided into filtering-based approaches [1,2,3,4,5] and graph optimization-based approaches [6,7,8,9,10,11]. With the maturity of feature tracking/matching techniques, feature-based approach has become a convention in visual-inertial algorithms. Most of these algorithms process image by tracking/matching sparse features, and minimize the reprojection error in the estimator [1,2,3,4,5,6,7,8,9,10]. Recently, direct approach draw researchers’ attention with its capability to exploit information from all intensity gradients in the image [12]. DSO (Direct Sparse Odometry), which came from Engel [13], showed remarkable performance in weak intensity variation environments. A tightly-coupled direct approach to visual-inertial odometry was proposed in [11] very recently, which can perform accurate and robust odometry estimation in little-texture or low-illumination environments. 

However, most methods assumed sensors are synchronized well under a common clock [1,2,4,5,6,7,8,9], and some of them also required the spatial parameters are determined exactly [1,5,6,7,8,9]. These requirements are not easy to be satisfied in practice. As a matter of fact, for most low-cost and self-assembled sensor suites, accurate factory calibration and hardware synchronization are not available. Consequently, these methods only work properly with a few well-calibrated and strictly-synchronized sensors.

In fact, sensor calibration for the spatial or temporal parameters has gathered tremendous research efforts. The observability of the spatial parameters is analyzed in [14,15], and the results show that the spatial parameters are observable given sufficiently excited motions. Four kinds of non-trivial degenerate motions for spatial-temporal calibration are studied in [16]. Furgale proposed a continuous-time batch optimization framework for spatial-temporal calibration [17], and provided a widely-used calibration toolbox, Kalibr. However, it requires artificial calibration objects and can only perform offline calibration. For online spatial calibration, Weiss considered optimizing the spatial parameters online in a nonlinear estimator [2]. Yang emphasized the importance of initial values for online calibration, and proposed initializing the spatial parameters together with the motion of system [18]. A similar initialization is performed in [19], where an iterative strategy is conducted to calibrate the extrinsic orientation and gyroscope bias. Nevertheless, these approaches did not consider the temporal offset. Moreover, Li proposed an approach to estimate motion with online temporal calibration in a multi-state constrained EKF framework. In our previous work, we studied the effect of the temporal offset on point reconstruction and proposed calibrating the temporal offset by shifting feature points to match IMU constraints [20]. A similar approach is performed in [21], where a coarse-to-fine strategy is applied to calibrate the temporal offset.

Among these calibration approaches, nearly all are built on feature-based visual-inertial odometry. For those approaches able to calibrate the temporal offset, the initialization for the temporal offset is not considered. Therefore, the online calibration may fail when a large temporal offset occurs.

**Figure 1:**
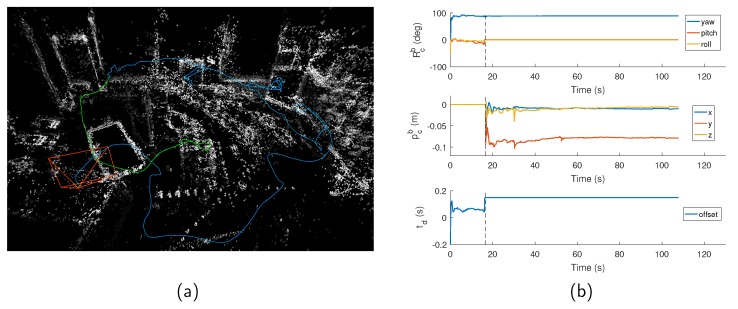
An illustration of performing our VIO algorithm on V2_01 easy (the camera delay set to 150 ms). (**a**) 3D reconstruction, camera trajectory (green line for VO, blue line for VIO), estimated pose (orange camera) at the end. (**b**) The spatial and temporal parameters estimated during the entire calibration process. The process can be divided into two stages: initialization and optimization, which are separated by dashed lines.

To this end, we implement a direct version of monocular VIO, and propose reliable initialization and online calibration for the spatial-temporal parameters. We assume the spatial-temporal parameters are constant but unknown variables. First, we perform VO (Visual Odometry) only. The spatial orientation and temporal offset are continuously estimated until they converge. After the initialization succeeds, the visual-inertial alignment is carried out to recover initial states for visual-inertial state estimation once excited motion is detected. Then, the visual-inertial odometry with online spatial-temporal calibration is launched. An illustration of performing our VIO algorithm is depicted in Figure 1. We highlight our contribution as follows:

- We design a feature-based initialization algorithm to initialize monocular direct visual odometry, which can detect motion effectively and initialize the map with higher robustness and efficiency compared to the initialization of DSO.
- We derive a robust and accurate optimization-based initialization to estimate the spatial orientation and temporal offset together. The initialization is able to recover sufficiently accurate results without any prior system knowledge or artificial calibration objects.
- We derive a monocular direct visual-inertial estimator with online spatial-temporal calibration. The estimator can also estimate other states such as IMU pose and 3D geometry.

## 2. Preliminaries

In this section, we describe the necessary notations for this paper, and give a definition for the spatial parameters and temporal offset. Besides, the error functions used in the nonlinear optimization are briefly reviewed.

### 2.1. Notation

In this paper, we use bold upper case letters A to represent matrices, bold lower case x to denote vectors. Scalars are represented by light lower case λ. We use quaternion q or rotation matrix R to denote rotation. If a vector/quaternion/rotation matrix describes the relative transformation from one original frame to another frame, a right subscript is appended to indicate the original frame, and the right superscript denotes the transformed frame, e.g., $p_{a}^{b}$ denotes the translation from frame *a* to frame *b*, $q_{a}^{b}$ or $R_{a}^{b}$ denotes the rotation from frame *a* to frame *b*. Moreover, we consider *v* as vision frame, which is defined by the first camera frame in visual odometry. We consider *w* as world frame, where gravity is along with *z* axis. We consider *b* as body frame aligned with IMU frame, and *c* as camera frame.

### 2.2. Spatial Parameters Definition

**Figure 2:**
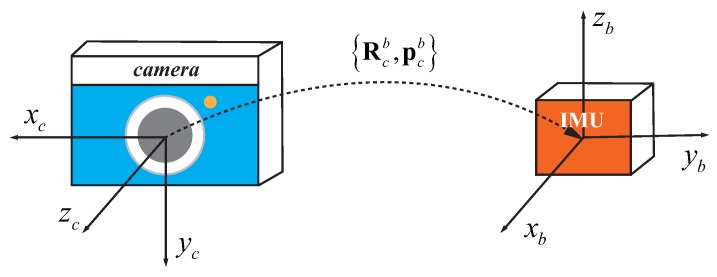
An illustration of the spatial parameters between IMU and camera.

To fuse IMU and camera measurements, the coordinate transformation between IMU and camera is required. In this paper, the spatial (extrinsic) parameters $\left\{R_{c}^{b},p_{c}^{b}\right\}$ between IMU and camera is the relative transformation from *c* to *b*, as illustrated in Figure 2.

### 2.3. Temporal Offset Definition

Timestamp of sensor measurements always suffers a delay, since the timestamp is generated after measurement creation. The delay has various causes: triggering delay, communication delay, unsynchronized clocks, etc. Here, we use *t* to denote measuring time, $t_{s}$ to denote timestamp, and $t_{delay}$ to denote the delay. The relationship of measuring time and timestamp is:

(1) $$t_{s}=t+t_{delay}$$

**Figure 3:**
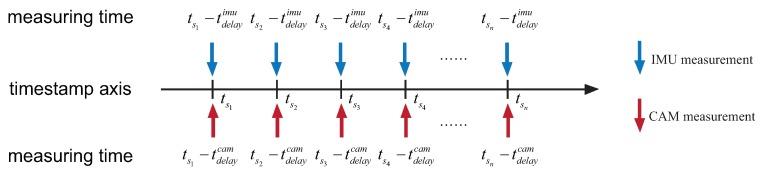
Misalignment of measurements: The IMU measurement and camera measurement with the same timestamp are not measured at the same time. In practice, we can only align sensor measurements according to their timestamps. However, it will result in a misalignment if these sensors suffer a different delay. To avoid this, we only need to shift the timestamp of one sensor from them (the camera or the IMU) with the temporal offset.

Therefore, if we directly align different sensors measurements with their timestamps, a temporal misalignment occurs, as illustrated in Figure 3. In this paper, we assume sensor delays are constant. Considering the IMU and camera measurements measured at the same time *t*, the timestamps of these measurements are:

(2) $$t_{s}^{imu}=t+t_{delay}^{imu},\;t_{s}^{cam}=t+t_{delay}^{cam}$$

The temporal offset can be defined as the difference of these two timestamps:

(3) $$t_{d}≜t_{s}^{cam}-t_{s}^{imu}$$

With this definition, we can align measurements with their timestamps easily. For example, given a camera image with a timestamp $t_{s}^{cam}$, the matching IMU measurement should have a timestamp $t_{s}^{cam}-t_{d}$. Conversely, given an IMU measurement with a timestamp $t_{s}^{imu}$, the image captured at the same time is attached with a timestamp $t_{s}^{imu}+t_{d}$.

### 2.4. Photometric Error

We use the same photometric error model as [13]; the photometric error of a point $p\in \Omega_{i}$ in host frame *i* reprojected in a target frame *j* is defined as:

(4) $$E_{p_{j}}≜\sum_{p\in N_{p}}w_{p}{\left|\left|\left(I_{j}\left[p^{\prime }\right]-b_{j}\right)-\frac{t_{j}e^{a_{j}}}{t_{i}e^{a_{i}}}\left(I_{i}\left[p\right]-b_{i}\right)\right|\right|}_{\gamma }$$

where $p_{j}$ is the point reprojected in frame *j*, p is a pixel from the pixels set $N_{p}$ of the point *p*, $w_{p}$ is the gradient-dependent weight of p, $p^{\prime }$ is the pixel reprojected into frame *j*, $I_{i}$ and $I_{j}$ are the image intensity of frame *i* and frame *j*, $t_{i},t_{j}$ are the exposure times, $a_{i},b_{i},a_{j},b_{j}$ are the illumination parameters and ${\left||\;\cdot \;|\right|}_{\gamma }$ is the Huber norm.

Then, we can formulate the total photometric error of all keyframes in the optimizing window as follows:

(5) $$E_{photo}=\sum_{i\in F}\sum_{p\in P_{i}}\sum_{j\in \mathrm{obs}(p)}E_{p_{j}}$$

where F is a set of keyframes in the window, $P_{i}$ is a set of sparse points in keyframe *i*, and obs(p) is a set of observations of the same point in other keyframes.

### 2.5. IMU Error

We follow the preintegration approach first proposed in [22] and extended by Forster [7], and we choose the quaternion-based derivation for our implementation [10]. This allows us to add IMU constraints between consecutive IMU states.

For two consecutive IMU states $s_{i}$ and $s_{i+1}$, after preintegration, we obtain an IMU preintegration measurement associated with a covariance matrix $\Sigma_{i,i+1}$. The IMU error function is

(6) $$E_{imu}\left(s_{i},s_{i+1}\right)≜r\left(s_{i},s_{i+1}\right)\Sigma_{i,i+1}^{-1}r\left(s_{i},s_{i+1}\right)$$

where $s_{i}≜{\left[{p_{b_{i}}^{w}}^{T},{q_{b_{i}}^{w}}^{T},{v_{b_{i}}^{w}}^{T},b_{a_{i}}^{T},b_{g_{i}}^{T}\right]}^{T}$, $p_{b}^{w}$ is IMU position, $q_{b}^{w}$ is IMU orientation, $v_{b}^{w}$ is IMU velocity, $b_{a}$ is accelerometer bias, $b_{g}$ is gyroscope bias, and $r(s_{i},s_{i+1})$ is the IMU preintegration residual defined in [10] (Equation (16)).

## 3. Methodology

This section details the proposed initialization and optimization for the spatial-temporal parameters. The system starts with direct visual odometry. During visual odometry, the system stores keyframe camera poses and corresponding IMU preintegrations, and then keeps initializing the spatial orientation and temporal offset by minimizing the rotation error between camera relative rotation and IMU pre-integrated rotation until a convergence threshold is exceeded. After the sensors are aligned spatially and temporally, the visual-inertial alignment is carried out to recover the scale, gravity and velocity for visual-inertial state estimation once excited motion is detected. Then, visual-inertial odometry is performed to optimize the spatial-temporal parameters together with IMU states and point inverse depths.

### 3.1. Initialize Monocular Direct VO

The monocular direct visual odometry proposed in [13] has shown high robustness and accuracy in motion tracking and 3D reconstruction, which inidicated the feasibility of using the poses from direct visual odometry to align with IMU preintegrations.

However, the initialization in [13] is slow and quite fragile, where map points are initialized by minimizing the photometric error directly. In fact, without any motion prior or structure prior, a corrupted map is likely to be created, which will reduce the accuracy and reliability of the following camera poses. Therefore, inspired by Mur-Artal et al. [23], we initialize visual odometry with a feature-based approach. We extract and track sparse features from the images. The camera poses and 3D points are recovered with two-view geometry constraints. Some direct approaches such as DTAM [24] also initialize with a feature-based approach. We highlight the difference between our initialization and the others: our initialization is more robust since we verify the translation of camera before the initialization is completed. Most feature-based initialization algorithms usually end up with a verification of the reprojection error, which is not reliable enough in our view because, in monocular visual odometry, the 3D structure is only able to be recovered properly with sufficiently translation. The steps of our algorithm are as follows:

1. Feature extracting:
  Extract sparse features [25] in the first frame, and record the amount *N* of features.
2. Feature tracking:
  Track features using KLT optical flow algorithm [26]. If the features amount $N_{c}<N\;\cdot \;T_{N}$, reset the first frame and go to Step 1.
3. Optical flow check:
  Measure camera motion by the root mean square optical flow $f=\sqrt{\frac{1}{n}\sum_{i=1}^{n}||p-p^{\prime }{\left|\right|}^{2}}$. If $f<T_{f}$, go to Step 2.
4. Motion recovery:
  Find the fundamental matrix F with feature correspondences and recover camera motion by decomposing F [27]. Then, triangulate points and check the reprojection error of the features to decide whether the recovery has succeeded or not. If the recovery fails, try to recover camera motion from the homography matrix H [27]. If both fail, go to Step 2. Otherwise, we can obtain the relative pose R,t from the first frame to the current frame, and the depth *d* of the features.
5. Translation verification:
  Warp the bearing vector of features with translation only $d_{t}^{\prime }p_{n,t}^{\prime }=d\;\cdot \;I_{3\times 3}p_{n}+t$, where $p_{n}$ is the bearing vector of p. Then, verify sufficient translation by checking the root mean square position offset $f_{t}=\sqrt{\frac{1}{n}\sum_{i=1}^{n}\left|\right|p_{n}-p_{n,t}^{\prime }{\left|\right|}^{2}}$. If $f_{t}<T_{f_{t}}$, go to Step 2.
6. Direct bundle adjustment and point activation:
  Perform direct bundle adjustment given the initial value of R,t and *d*, to refine the initial reconstruction and estimate the relative illumination parameters from the first frame to the current frame. Then, extract more points on the first frame, and do a discrete search on epipolar line to activate these candidates for the following visual odometry.

**Figure 4:**
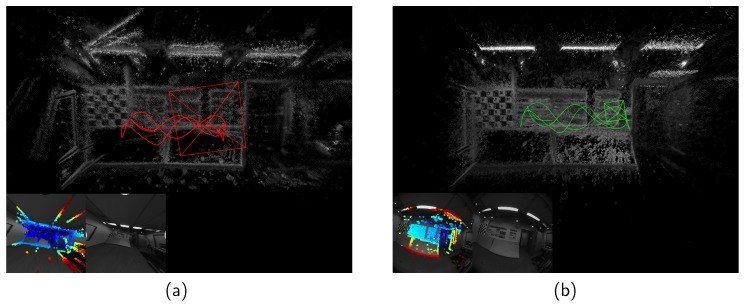
Visual odometry on Room 1. (**a**) DSO, which initializes VO with a direct approach. Notice the top left part of the recovered 3D geometry is incorrect. (**b**) Our method that initializes visual odometry with a feature-based approach. The 3D reconstruction is more proper.

An example of VO initialization on Room 1 [28] is shown in Figure 4. It is obvious that DSO generated a corrupted map after initialization, while the structure was recovered correctly with our feature-based initialization algorithm.

### 3.2. Initialization for Spatial-Temporal Parameters

Considering two consecutive frames *i* and i+1, we get the camera rotation $q_{c_{i}}^{v}$ and $q_{c_{i+1}}^{v}$ from visual odometry, as well as the preintegrated rotation ${\tilde{\gamma }}_{b_{i+1}}^{b_{i}}$ from IMU preintegration. We can establish an equation of rotation residual as follows:

(7) $$r_{i,i+1}=2\;\cdot \;\mathrm{Vec}\left[{q_{c}^{b}}^{-1}⊗{\hat{\gamma }}_{b_{i+1}}^{b_{i}}⊗q_{c}^{b}⊗{q_{c_{i+1}}^{c_{i}}}^{-1}\right]$$

where $q_{c_{i+1}}^{c_{i}}={q_{i}^{v}}^{-1}⊗q_{i+1}^{v}$, ${\hat{\gamma }}_{b_{i+1}}^{b_{i}}\approx {\tilde{\gamma }}_{b_{i+1}}^{b_{i}}⊗\left[\begin{array}{c} 1\\ J_{b_{g}}^{\gamma }\delta b_{g} \end{array}\right]$, and Vec[q] is the vector part of the quaternion q. Actually, we can estimate the gyroscope bias $b_{g}$ and the extrinsic rotation $q_{c}^{b}$ together by solving a nonlinear least square problem with the rotation residuals constructed from all stored keyframes, if there is no temporal offset.

However, there may be a temporal misalignment between the IMU preintegrated rotation and the camera relative rotation. Assume ${\tilde{\gamma }}_{b_{i+1}}^{b_{i}}$ is calculated from the IMU measurements with timestamps between $t_{s_{i}}^{imu}$ and $t_{s_{i+1}}^{imu}$. $q_{c_{i+1}^{\prime }}^{c_{i}^{\prime }}$ is the relative rotation of two camera poses with the same timestamps $t_{s_{i}}^{imu}$ and $t_{s_{i+1}}^{imu}$. According to the definition of the temporal offset (Equation (3)), to align the camera poses to the IMU preintegrated rotation, the timestamps of the matched images are $t_{s_{i}}^{cam}=t_{s_{i}}^{imu}+t_{d}$ and $t_{s_{i+1}}^{cam}=t_{s_{i+1}}^{imu}+t_{d}$. Therefore, the aligned relative camera rotation is

(8) $$q_{c_{i+1}}^{c_{i}}=q_{c_{i}^{\prime }}^{c_{i}}q_{c_{i+1}^{\prime }}^{c_{i}^{\prime }}q_{c_{i+1}}^{c_{i+1}^{\prime }}$$

Assuming the camera rotates in a constant angular velocity between two keyframes, we can get $q_{c_{i}^{\prime }}^{c_{i}}\approx \left[\begin{array}{c} 1\\ -\frac{1}{2}\omega_{c_{i}^{\prime }}^{c_{i-1}^{\prime }}\;\cdot \;t_{d} \end{array}\right],q_{c_{i+1}}^{c_{i+1}^{\prime }}\approx \left[\begin{array}{c} 1\\ \frac{1}{2}\omega_{c_{i+2}^{\prime }}^{c_{i+1}^{\prime }}\;\cdot \;t_{d} \end{array}\right]$, where $\omega_{c_{i}^{\prime }}^{c_{i-1}^{\prime }},\omega_{c_{i+2}^{\prime }}^{c_{i+1}^{\prime }}$ are camera angular velocities that can be calculated from the stored keyframe poses as follows:

(9) $$\omega_{c_{i}^{\prime }}^{c_{i-1}^{\prime }}=\frac{2\;\cdot \;\mathrm{Vec}[q_{c_{i}^{\prime }}^{c_{i-1}^{\prime }}]}{t_{s_{i}}^{imu}-t_{s_{i-1}}^{imu}},\;\omega_{c_{i+2}^{\prime }}^{c_{i+1}^{\prime }}=\frac{2\;\cdot \;\mathrm{Vec}[q_{c_{i+2}^{\prime }}^{c_{i+1}^{\prime }}]}{t_{s_{i+2}}^{imu}-t_{s_{i+1}}^{imu}}$$

By substituting $q_{c_{i+1}}^{c_{i}}$ of Equation (8) into Equation (7), we can estimate the extrinsic rotation, temporal offset and gyroscope bias jointly by minimizing the following error function:

(10) $$E_{rot}=\sum_{i\in F_{a}}{\left|\left|r_{i,i+1}\right|\right|}_{\gamma }$$

where $F_{a}$ is a set of all stored keyframes. We do not consider initializing the extrinsic translation since it is usually small and can be simply initialized to $0_{3\times 1}$ in practice.

### 3.3. Visual-Inertial Nonlinear Optimization

After initializing the spatial and temporal parameters, we perform a loosely coupled approach proposed in [29] to recover the velocity, gravity and metric scale. Then, we can launch a tightly coupled estimator to optimize all states jointly. For each active keyframe, we define a state vector (the transpose is ignored for states definition in Equations (11) and (12))

(11) $$x_{i}=\left[s_{i},a_{i},b_{i},\lambda_{i}^{1},\lambda_{i}^{2},\ldots ,\lambda_{i}^{m}\right]$$

where $s_{i}$ is the IMU state defined in Section 2.5. $a_{i},b_{i}$ are the illumination parameters, and $\lambda_{i}^{k}$ is the inverse depth of the *k*th point hosted in the *i*th keyframe.

The full states of optimization are defined as follows:

(12) $$x=[x_{1},x_{2},\ldots ,x_{n},p_{c}^{b},q_{c}^{b},t_{d}]$$

We assume the IMU in the system is moving with a constant velocity during a short period of time. Thus, the IMU pose at any time can be extrapolated with its nearest IMU pose, linear velocity and angular velocity, which means

(13) $$p_{b}^{w}\left(t\right)\approx p_{b}^{w}\left(t_{0}\right)+v_{b}^{w}\left(t_{0}\right)\;\cdot \;\left(t-t_{0}\right),\;R_{b}^{w}\left(t\right)\approx R_{b}^{w}\left(t_{0}\right)\left[I+{\left[\omega \left(t_{0}\right)\right]}_{\times }\;\cdot \;\left(t-t_{0}\right)\right]$$

where ω is the angular velocity of the IMU. With Equation (13), we can calculate the IMU poses at the time when the images are captured.

(14) $$\begin{array}{c} p_{b_{i}^{\prime }}^{w}\approx p_{b_{i}}^{w}-v_{b_{i}}^{w}t_{d},\;R_{b_{i}^{\prime }}^{w}\approx R_{b_{i}}^{w}\left(I-{\left[\omega_{i}\right]}_{\times }t_{d}\right) \end{array}$$

Thus, considering the spatial-temporal parameters, the reprojection formula can be written as

(15) $$\begin{array}{c} p^{\prime }\left(p_{c}^{b},q_{c}^{b},t_{d}\right)=\Pi \left\{\rho_{j}{R_{c}^{b}}^{T}\left[{R_{b_{j}^{\prime }}^{w}}^{T}\left(R_{b_{i}^{\prime }}^{w}\left(R_{c}^{b}\rho_{i}^{-1}\Pi^{-1}\left(p\right)+t_{c}^{b}\right)+p_{b_{i}^{\prime }}^{w}-p_{b_{j}^{\prime }}^{w}\right)-t_{c}^{b}\right]\right\} \end{array}$$

where Π(·) is the projection function, which projects a 3D point into the pixel plane. $\Pi^{-1}\left(\;\cdot \;\right)$ is the back projection function, which turns a pixel into a bearing vector using camera intrinsic parameters. With Equation (15), we can evaluate the photometric error with IMU pose, velocity, point inverse depth and the spatial-temporal parameters.

It should be noted that we ignore the visual constraints on IMU velocity to reduce the computation complexity, and no notable effect on accuracy is observed. All states are optimized by minimizing the object function

(16) $$\min_{x}\left\{E_{photo}+E_{imu}+E_{prior}\right\}$$

where $E_{photo}$ and $E_{imu}$ are the IMU error and the photometric error defined in Section 2.4 and Section 2.5, respectively. The prior error $E_{prior}$ is evaluated from the prior information, and the prior is obtained by marginalizing past states using the Schur complement [30] with the two-way marginalization strategy proposed in [31]. To maintain consistency of the estimator and reduce computational complexity, we apply the “First estimate Jacobians” (FEJ) approach proposed in [32], which means all states constrained by the prior in the sliding window are linearized at the same point as in previous marginalization. We solve the nonlinear least square problem with the Levenberg–Marquardt (L-M) method.

### 3.4. Criteria in Initialization and Optimization

To perform robust initialization and calibration for the spatial-temporal parameters, several criteria should be met to ensure all procedures perform properly. The initialization should end up with a convergence criteria satisfied. The online calibration is required to begin with sufficiently excited motion, otherwise the system matrix will suffer rank-deficiency due to the unobservable states. 

**(1) Initialization termination criteria**

Successful calibration of the spatial-temporal parameters (exclude extrinsic translation) and gyroscope bias relies on the observability of these states. Under good observability, the null space of the Jacobian for Equation (7) should be rank one. Therefore, we detect the observability of the states by checking whether the second smallest singular value of the Jacobian $\sigma_{J}^{\min_{2}}$ is sufficiently large. If $\sigma_{J}^{\min_{2}}>T_{\sigma }$, these states are possible to be identified.

Additionally, we check the average rotation error to make sure the states are estimated correctly. The average rotation is defined as follows:

(17) $$A_{rot}=\sqrt{\frac{E_{rot}}{M}}$$

where *M* is the number of all stored keyframes used in initialization. The initialization process terminates if $\sigma_{J}^{\min_{2}}>T_{\sigma }$ and $A_{rot}<T_{A}$. 

**(2) Sufficient excitation condition**

Before performing the online calibration, we need to check whether the motion is excited enough. According to the study in [16], under several types of degenerate motion, the spatial-temporal parameters are not able to be determined completely. Thus, we verify the excitation by checking whether the variance of the spatial-temporal parameters is sufficiently small. The covariance of full states is the inverse Hessian matrix of the states.

(18) $$\mathrm{Cov}\left(x\right)=H^{-1}$$

After the nonlinear estimator is launched, we do not optimize the spatial-temporal parameters immediately. We set three thresholds for the variance of the extrinsic rotation, translation and temporal offset, respectively. The specific parameters are only estimated after the corresponding variance is lower than its threshold. For example, if $\mathrm{Var}\left(p_{c}^{b}\right)<T_{p_{c}^{b}}$, we start to estimate $p_{c}^{b}$ in the estimator. Before that, $p_{c}^{b}$ is fixed by setting the corresponding columns of the Jacobians of the residual vector to zero.

## 4. Experimental Results

We verified the performance of our initialization and online calibration with the EuRoC dataset [33]. The dataset provides stereo images (Aptina MT9V034 global shutter, 20 FPS), synchronized IMU measurements (ADIS16448, 200 Hz) and ground truth states (Leica MS50 and VICON). We only used the left camera from stereo images set. To demonstrate the capability of spatial-temporal estimation, we first set the temporal offset by manually shifting image timestamps with a constant camera delay and generated time-shifted sequences. Then, we tested the proposed algorithm and other methods on these sequences with the initial values of $\left\{R_{c}^{b},p_{c}^{b},t_{d}\right\}$ set to $\left\{I_{3\times 3},0_{3\times 1},0\right\}$. All experiments were carried out on a laptop computer with Intel CPU i7-3630QM (4 cores @2.40 GHz) and 16 GB RAM. The parameters we mentioned above were set as follows: $T_{N}=0.6$, $T_{f}=100$, $T_{f_{t}}=0.025$, $T_{A}=1.2$, $T_{\sigma }=0.05$, $T_{p_{c}^{b}}=10^{-4}$, $T_{q_{c}^{b}}=10^{-3}$, and $T_{t_{d}}=10^{-7}$. In our experience, these parameters can be set in a wide range and have no significant impact on the performance. It should also be noted that we evaluated the orientation error and translation error using the following formulas, respectively:

(19) $$e_{\mathrm{orien}}=\sqrt{e_{\mathrm{yaw}}^{2}+e_{\mathrm{pitch}}^{2}+e_{\mathrm{pitch}}^{2}},\;e_{\mathrm{trans}}=\sqrt{e_{x}^{2}+e_{y}^{2}+e_{z}^{2}}$$

### 4.1. Spatial-Temporal Initialization Performance

**Figure 5:**
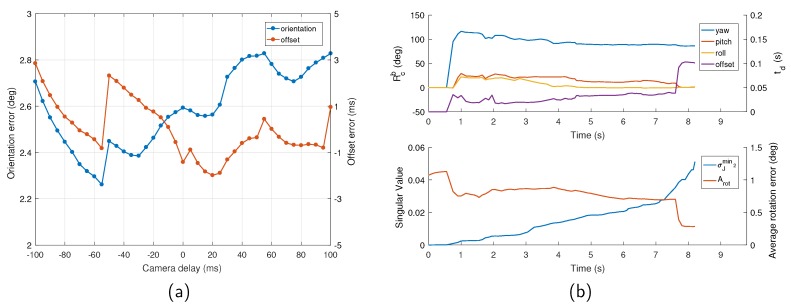
Spatial-temporal initialization results on V1_02_medium. (**a**) The orientation and time offset errors with respect to the different predefined camera delays. All the orientation errors are below three degrees and all the temporal offset errors are lower than 3 ms, which proves our approach is able to recover accurate enough orientation and offset under a wide range of temporal offsets. (**b**) Detailed illustration of the entire initialization process when the camera delay is set to 100 ms. (**Top**) The spatial orientation and time offset estimated. (**Bottom**) The second smallest singular value and the average rotation error.

In this test, the sequence V1_02_medium was used to verify the performance of the proposed initialization. To demonstrate the capability of our approach under different temporal offsets, we set the camera delays from −100 to 100 ms manually, and tested these time-shifted sequences with our initialization method. The result is depicted in Figure 5a. It can be seen that the initialization could obtain accurate extrinsic orientation and temporal offset for a wide range of temporal offsets, achieving a typical precision of 3 degrees for the orientation and 3 ms for the time offset, which are sufficiently fine to support the following optimization.

Typical time varied characteristic curves of the spatial-temporal parameters and the convergence criteria are shown in Figure 5b. It is evident that, over time, $\sigma_{J}^{\min_{2}}$ became larger due to the accumulated measurements, which indicates the growing observability of the orientation and offset. Additionally, the orientation gradually converged and could be determined well even when the offset was quite inaccurate. On the contrary, the accuracy of the time offset estimate was highly dependent on the observability of the system (i.e., whether $\sigma_{J}^{\min_{2}}$ was sufficiently large). Only when $\sigma_{J}^{\min_{2}}$ exceeded a certain threshold, the temporal offset was immediately estimated at high accuracy, and the average rotation error decreased instantly, which proved the necessity and feasibility of the proposed criteria.

### 4.2. Overall Performance

We next compared our method against VINS-Mono [10], which is another state-of-the-art visual-inertial odometry algorithm with online spatial-temporal calibration ability. To test the performance under different time offsets, we set the camera delay to 0 ms, 50 ms and 100 ms on 11 EuRoC datasets, and launched the programs on these time-biased sequences. The VINS-Mono was launched without knowing the prior spatial-temporal parameters. The errors of the calibrated spatial-temporal parameters and the absolute translational RMSE (Root Mean Square Error) of the keyframe trajectory are shown in Table 1. All of these results are the median over five executions in each sequence.

It can be seen that our method was more robust against large temporal offset, since we determined the offset in the initialization. The temporal offset estimated with our method achieved sub-millisecond accuracy, which was much more accurate than the offset estimated from VINS-Mono. It can be interpreted as having two reasons: (1) we initialized and obtained a accurate temporal offset before the nonlinear optimization, while VINS-Mono directly estimated the offset during the nonlinear optimization linearizing at an inaccurate time offset; and (2) to match visual measurements to IMU constraints, we extrapolated IMU pose with instant IMU state and measurement for visual point reprojection, while VINS-Mono extrapolated feature position with average camera motion. The average camera motion was of lower accuracy than instant IMU state, especially when the system was in high dynamic environments. Both VINS-Mono and our method could estimate extrinsic orientation and translation with errors of about 0.6 degrees and 0.02 m, respectively. In terms of the trajectory accuracy, most of the trajectories estimated by our method were of higher accuracy than those of VINS-Mono, especially on the MH sequences.

## 5. Conclusions

In this paper, we perform a direct version of monocular visual-inertial odometry, and propose a novel initialization and online calibration for the spatial-temporal parameters without any prior information. Specifically, our approach is able to automatically identify observability and convergence of the spatial-temporal parameters. We highlight that our approach is a general model, and can be easily adopted into either direct-based or feature-based VIO frameworks. Experiments demonstrated that our approach achieves competitive accuracy and robustness compared with the state-of-the-art approach, especially when the temporal offset is large.

Moreover, our approach can be extended for rolling shutter calibration. Considering most smart devices (e.g., smartphones and tablets) choose rolling shutter cameras to capture images for the cheaper price and the potentially higher frame rate and resolution than global shutter cameras, rolling shutter calibration is essential for the visual-inertial odometry using a smart device. We plan to extend our approach on rolling shutter cameras next.

## Figures and Tables

**Table 1:** Spatial-temporal calibration error and keyframe trajectory accuracy.

Sequence	Camera Delay (ms)	Ours				VINS-Mono			
		$e_{\mathrm{orien}}$ (${}^{\circ }$)	$e_{\mathrm{trans}}$ (m)	$e_{\mathrm{offset}}$ (ms)	RMSE (m)	$e_{\mathrm{orien}}$ (${}^{\circ }$)	$e_{\mathrm{trans}}$ (m)	$e_{\mathrm{offset}}$ (ms)	RMSE (m)
V11	0	0.583	0.022	**−0.15**	**0.073**	**0.566**	**0.020**	−1.52	0.096
	50	0.588	0.023	**−0.21**	**0.073**	**0.571**	**0.016**	−1.77	0.084
	100	**0.577**	0.022	**−0.15**	0.077	0.624	**0.010**	−3.23	**0.067**
V12	0	0.563	**0.019**	**−0.09**	0.118	**0.534**	0.046	−0.57	**0.091**
	50	**0.559**	0.019	**−0.10**	0.116	0.623	**0.018**	−0.88	**0.070**
	100	**0.569**	0.021	**−0.10**	0.143	0.672	**0.018**	−1.53	**0.064**
V13	0	**0.507**	**0.013**	**−0.33**	**0.118**	0.515	0.017	−0.35	— ${}^{1}$
	50	**0.508**	0.016	**−0.33**	**0.121**	0.547	**0.010**	−0.87	0.407
	100	**0.513**	**0.014**	**−0.39**	**0.093**	—	—	—	—
V21	0	0.491	**0.023**	**−0.33**	0.099	**0.471**	0.024	−1.11	**0.065**
	50	**0.457**	0.025	**−0.29**	0.088	0.573	**0.021**	−0.95	**0.053**
	100	**0.513**	0.022	**−0.36**	0.082	0.645	**0.019**	−2.32	**0.034**
V22	0	**0.553**	0.020	**−0.09**	0.099	0.599	**0.014**	−0.40	**0.090**
	50	**0.558**	0.020	**−0.09**	**0.089**	0.651	**0.013**	−0.49	0.144
	100	**0.558**	0.020	**−0.09**	**0.100**	0.581	**0.009**	−0.79	—
V23	0	**0.633**	**0.015**	**−0.09**	**0.135**	0.640	0.016	−0.38	0.146
	50	**0.626**	0.015	**−0.04**	0.234	0.658	**0.014**	−0.55	**0.114**
	100	0.633	**0.014**	**−0.04**	0.233	**0.609**	0.016	−0.74	**0.128**
MH1	0	**0.501**	0.018	**−0.16**	**0.080**	0.552	0.018	−0.68	0.241
	50	**0.505**	0.015	**−0.12**	**0.119**	0.556	**0.014**	−0.85	0.247
	100	**0.481**	**0.015**	**−0.12**	**0.111**	0.533	0.025	−1.49	0.366
MH2	0	0.621	0.014	**−0.29**	**0.082**	**0.537**	**0.010**	−0.93	0.292
	50	0.624	0.014	**−0.34**	**0.086**	**0.512**	**0.008**	−1.25	0.277
	100	0.634	0.015	**−0.21**	**0.074**	**0.556**	**0.014**	−1.05	—
MH3	0	0.619	0.022	**−0.01**	**0.161**	0.619	**0.019**	−0.82	0.192
	50	**0.627**	0.024	**−0.05**	**0.133**	0.671	**0.014**	−1.20	0.189
	100	**0.607**	**0.020**	**−0.09**	**0.173**	1.132	0.035	−2.77	—
MH4	0	**0.554**	**0.019**	**0.11**	**0.197**	0.560	0.022	−1.15	0.372
	50	**0.521**	0.013	**0.17**	**0.178**	0.558	0.013	−1.46	0.487
	100	0.512	0.018	**−0.03**	**0.143**	**0.468**	**0.007**	−3.12	0.331
MH5	0	0.605	**0.013**	**−0.09**	**0.162**	**0.538**	0.020	−1.26	0.309
	50	**0.509**	**0.010**	**−0.20**	**0.207**	0.547	0.017	−1.49	0.299
	100	0.552	**0.017**	**−0.17**	**0.205**	**0.435**	0.088	−2.20	1.141

${}^{1}$ “—” means that the tracking fails at some time, and the result is of huge error.
